# When the clinic becomes a home. Successful VCT and ART services in a stressful environment

**DOI:** 10.1080/17290376.2016.1222301

**Published:** 2016-09-06

**Authors:** Jonathan Mensah Dapaah, Rachel Spronk

**Affiliations:** ^a^ is a lecturer at the Sociology and Social Work Department, Kwame Nkrumah University of Science and Technology, Kumasi, Ghana; ^b^ is assistant professor at the Sociology and Anthropology Department, University of Amsterdam, Amsterdam, The Netherlands

**Keywords:** ART clinics, HIV/AIDS stigma, clients, health workers, therapy management group, clinique ART, VIH/SIDA stigmate, clients, d'agents de santé, groupe de gestion therapeutique

## Abstract

With the upscaling of antiretroviral therapy (ART) in resource-poor countries, many HIV-positive persons in Ghana have been accessing treatment in hospitals. Prevalence is relatively low compared to other African countries, 1.30%. HIV/AIDS remains heavily stigmatised in Ghana, which influences the provision and use of ART. This article investigates how HIV-positive persons accessing care and treatment go about their everyday lives in the ART clinic and how they have eventually come to see the clinic as a safe place that they call ‘home’. The study took place in two Ghanaian hospitals in the Ashanti Region which in 2013 had the country’s highest HIV prevalence rate of 1.30% [Ghana Health Service [GHS]/National AIDS Control Programme [NACP] (2013). 2013 HIV Sentinel Survey Report, Accra, Ghana]. It was conducted through ethnographic research, with data gathered in the two facilities through participant observation, conversations and in-depth interviews. It took place over a period of 15 months, between 2007 and 2010. In all, 24 health workers and 22 clients were interviewed in depth, while informal conversations were held with many others. The findings show that clients have adopted the clinic as a second home and used it to carry out various activities in order to avoid identification and stigmatisation as People Living with AIDS (PLWA). The most dramatic outcome was that, contrary to Ghanaian norms and values, people turned to non-kin for assistance. Accordingly, fellow clients and health personnel, rather than relatives, have become their ‘therapy management group’ [Janzen, J. M. (1987). Therapy Management: Concept, Reality, Process. Medical Anthropology Quarterly, 1(1), 68–84]. The clients have thus created a fictive family within the clinic – made up of health workers (as ‘parents’), the clients themselves (as ‘children’) and the peer educators (as ‘aunts’ and ‘uncles’). In the face of persistent stigma associated with HIV infection in Ghana, the use of the clinic as a ‘home’ has on the one hand helped those receiving treatment to maintain their position, respect and reputation within their families and community, while on the other it prevents PLWA from disclosing. The study concludes that compassion is an important element in the professionalisation of healthcare workers in low-prevalence countries.

## Résumé

Avec l’élargissement de la thérapie antirétrovirale dans des pays pauvres en ressources, beaucoup de personnes qui vivent avec le VIH ont actuellement accès au traitements dans des hôpitaux. Au Ghana, comparé aux autres pays africains, la prévalence de VIH est relativement faible: 1,30 pourcent. Néanmoins, le VIH/SIDA y reste sérieusement stigmatisé, ce qui affecte la provision et l’utilisation des thérapies antirétrovirales (ARV). Cet article montre comment les personnes vivant avec le virus qui accèdent les soins et les traitements mènent leur vie quotidienne dans des cliniques ART et comment elles arrivent à considérer la clinique comme un espace sûr qu’elles appellent leur « chez-soi ». Cette étude a eu lieu pendant 15 mois entre 2007 et 2010 dans deux hôpitaux ghanéens dans le Région Ashanti. Dans cette enquête ethnographique, on a généré nos données à travers l’observation participante dans les deux établissements et à travers des conversations et des entretiens approfondis. En total, 24 agents de santé et 22 clients ont été interviewés, alors que des conversations informelles ont eu lieu avec les patients, les agents et les pairs éducateurs. Nos observations démontrent que les clients ont adopté la clinique comme un deuxième chez-soi et l’utilisaient pour y exercer des activités diverses, afin d’éviter l’identification et la stigmatisation comme des « personnes vivant avec le SIDA » (PLWA – People Living With AIDS). Le résultat le plus frappant était que, contrairement au normes et valeurs ghanéens, les clients se tournaient d’avantage vers des non-parents pour trouver de l’assistance. Plutôt que des parents, ce sont donc les autres clients et les agents de santé qui constituaient le « groupe de gestion thérapeutique » (Janzen 1987). Les clients créent ainsi une famille fictive au sein de la clinique – composée d’agents de santé (les « parents »), des clients eux-mêmes (les « enfants ») et des pairs éducateurs (les « tantes » et les « oncles »). Dans le contexte de la stigmatisation tenace du VIH au Ghana, l’utilisation de la clinique comme un chez-soi aide – d’une part – ceux qui reçoivent des traitements à maintenir leur position, leur respect et leur réputation dans leurs familles et communautés, alors que – d’autre part – cette stratégie évite également l’exposition des personnes vivant avec le SIDA. Nous concluons donc que la compassion est un élément important dans la professionnalisation des agents de santé dans des pays à un taux de prévalence relativement faible.

## Introduction

Stigma and discrimination relating to HIV/AIDS continue to undermine public health efforts to contend the epidemic. Stigma negatively affects preventative behaviours, HIV test-seeking behaviour, care-seeking behaviour on diagnosis, quality of care provided to HIV-positive persons, and perception and treatment of persons living with HIV/AIDS by communities, families and partners. The literature discusses three main forms of stigma: perceived, enacted and self-stigma (cf. Brown, Macintyre & Trujillo 2003). Most studies on stigma and HIV/AIDS in Africa focus on the way people anticipate and deal with stigma in their families and communities (cf. Mbonu, Van Den Borne & De Vries 2009; cf. Stangl, Lloyd, Brady, Holland & Baral 2013) and on the effectiveness of HIV-related interventions (cf. French, Greeff & Watson 2014; cf. Sengupta, Banks, Jones, Miles & Smith 2011). A frequent focus of study is the issue of how stigma inhibits the uptake of antiretroviral therapy (ART) (cf. Mall, Middelkoop, Mark, Wood & Bekker 2013). Less attention has been given to how those receiving treatment strategise and use health facilities to conceal their status as a way of dealing with stigma. This article is about how clients^1^ in Ghana go about their everyday life in the ART clinic during care and treatment, in a context of severe AIDS stigma (Agyemang 2013; Kwansa 2013; Riley & Baah-Odoom 2012).

The article explores the ways in which those undergoing treatment in two specific clinics have created a private place out of a public space and how they use it for various activities in such a way as to keep their HIV status hidden. The clients have thus adopted the health workers as their ‘family members’ and the clinic as a ‘home’ that operates in tandem with their real homes in the community. Consequently, this new-found family has come to serve as their ‘therapy management group’, a term coined by Janzen (1987). The therapy management group is a special-purpose group made up of those who are closely associated with the person receiving treatment – they constitute a fictive ‘kin group’ involved with the sufferer in order to lend assistance or assume authority in providing a diagnosis and therapy.

This finding stands in contrast to a general perception in sub-Saharan Africa that health workers, particularly nurses, often do not treat patients or clients well (Van der Geest & Sarkodie 1998; cf. Jewkes, Abrahams & Mvo 1998). There is a perception that health workers are rude and harsh towards clients in general, while giving immediate and high-quality treatment to those who are personally known to them. According to Andersen (2004), in their treatment of patients in a Ghanaian hospital health workers distinguished between class or education level. Andersen reports that uneducated clients in particular, disparagingly called ‘villagers’, were treated with impatience and discourtesy, given less information and accorded less time; they were ordered around, yelled at and accused of lying. Indeed, presently in Ghana, there is a widespread popular idea that health workers are harsh and frequently treat patients with no respect. However, contrary to this prevalent stereotyping of healthcare workers, the most important finding of this study is that most nurses practise professionally and with compassion. The nurses in this study acted as advisors to clients on a wide range of issues besides treatment and care. In short, they took care of clients and, in the words of those receiving the service, made them feel at home.

In Ghana, hospitals are considered to be uncomfortable if not dangerous and hostile locations. The majority of Ghanaians, rather than being admitted to a hospital, are usually interested in simply getting treatment and then to go home to recover. However, if a patient’s sickness is serious and needs close monitoring, they do generally agree to be admitted to a ward. But the fact remains that Ghanaians are ambivalent about hospital attendance; they seek healthcare, but somewhat reluctantly. By contrast, the clients in the ART clinics in this study had a strong attachment to the facilities where they received care and treatment. This observation is in line with the findings of Wilson and Luker (2006) where stigmatised cancer patients found a place of shelter or seclusion, in their words ‘a home’, in the hospital where they were treated wholly as people rather than merely as a negative function of their affliction. Similarly, clients in this HIV/AIDS study often feared stigmatisation and as a result, they started using the clinic as a place where they could escape from public view and feel at home without having to be constantly vigilant about their HIV status. Since the treatment they received is lifelong, it is important to understand why and how the clinic becomes a safe place and how, ironically, it enables clients to prevent and/or delay the disclosure of their status to their environment.

How then did clients gradually get accustomed to the clinic? This article examines three main issues. It starts with an analysis of why the family homes of clients are no longer experienced as homes, followed by a discussion of what makes the clinic a home for clients, and concluding that the fictive kin relations in the clinic function as an alternative to the domestic therapy management group. In this regard, the use of the clinic as a home has some parallels with the Creativity Initiative, which was instituted in a clinic in Uganda to help reduce HIV-related stigma (Neema, Atuyambe, Otolok-Tanga, Twijukye, Kambugu, Thayer, *et al*. 2012). This study explains how empowering people with HIV or AIDS (PLWA) in sometimes unexpected ways helps them to deal with stigma.

## Setting and study design

The study on which this article is based is part of a larger research project studying the upscale of ART and voluntary counselling and testing (VCT) in Ghana, made up of three interrelated studies on the provision and use of VCT and ART. The overall research project conducted an in-depth investigation of the social and cultural factors that discourage as well as encourage HIV-positive persons and other people from using VCT and ART services. It focused on the clinic environment and on the means by which health workers provide VCT and ART, as well as on the perceptions and approaches of health workers towards clients, and vice versa. The first study (and the focus in this article) was carried out in two health facilities providing VCT and ARV therapy, and examined how conditions within two institutions influenced the use of counselling and testing services (Dapaah 2012). The second study was conducted in the communities served by the two hospitals in which the first study was carried out. It examined the clients’ points of view regarding VCT and ART and the way the services are provided through medical channels (Kwansa 2013). Social and cultural perceptions of blame, shame and stigma took a central place in both research projects. The third study considered the policy-making processes at international, national and local levels (Spronk 2011).

The two hospitals that feature in this article were among the few health facilities in the region that benefited from the initial scale-up of VCT and ART from 2004 to 2009. One hospital is a larger public hospital in a main city serving a population of more than 50,000, while the other is a smaller hospital is a rural area. For purposes of anonymity, the names of the two hospitals will not be mentioned. The estimated HIV prevalence rate of this region was 3.2% in 2013. This figure is higher than the national rate of 1.5% and is the fourth highest out of the country’s 10 regions (GHS/NACP 2013). At the end of 2013, there were 324 VCT centres and 26 ART clinics in the region.

The study is based on a hospital ethnography which had the healthcare setting as the ‘field’ for data collection (Van Der Geest & Finkler 2004). The key researcher and first author spent a total of 15 months collecting data in the hospitals between August 2007 and January 2010. The central research question considered how the attitudes and behaviours of health workers influenced the provision and use of voluntary counselling and testing and ART treatment. The data were compiled successively from participant observation to informal conversations and in-depth interviews. These were supplemented with analyses of hospital records and the socio-demographic characteristics of clients. The trademark of ethnography, participant observation, takes a particular role in hospital ethnography where the emphasis comes to lie on observation (Wind 2008). As such, the methodological focus of this study was on clinical interactions between patients and health workers, including undergoing medical procedures and engaging in sometimes emotionally disturbing conversations about what decisions to make concerning the handling, caring and treatment of the disease. Particularly, the researcher participated in counselling sessions and observed the interactions between counsellors and clients. He also engaged in conversations with health workers and clients to get their views on provision and use of services. Unlike interviews, which were considered formal by health workers and clients, these conversations were less likely to inhibit participants from speaking out.

More than 40 health workers in the VCT centres and ART clinics were observed during data collection. Purposive sampling was used to select health workers for interviews based on the following set of criteria: (1) those who had most contact with clients on a daily basis; (2) those who provided VCT or ART; (3) the position the health worker occupies in the clinic; and (4) his/her willingness to be interviewed. The selection of clients was based on their health condition (for example, when they were not too ill and psychologically traumatised by HIV infection) and on their willingness to be interviewed. In total, the group of health workers and clients roughly represented the general populations of health workers and HIV/AIDS clients in terms of age, gender, ethnicity and religion. In all, 24 health workers who provided counselling, testing and treatment were interviewed. The group was made up of six nurses, five medical doctors, one pharmacist, one pharmacist technologist, two laboratory technicians, one disease control officer, five counsellors, one health assistant and two cleaners. Twenty-two clients were interviewed, comprising 12 women and 10 men. More female than male clients were interviewed because the majority of clients were female, which confirmed the gender difference by the hospital records.

Ethical clearance for the research was sought from the Ethics Committees of Ghana Health Service under the Ghana Ministry of Health, and from the two hospitals. Consent for participation was received from health workers and clients of the VCT centres and ART clinics. The study participants were assured of anonymity in presentations and publications. As a result, all the names used in the article are fictitious.

## HIV/AIDS stigma

In Ghana, HIV prevalence is relatively low compared to that in other sub-Saharan African countries; at 1.30% in 2013 (GHS/NACP 2013). Numbers of people living with HIV/AIDS (PLWHA) are therefore relatively small and not many Ghanaians know somebody who is infected. HIV/AIDS is heavily stigmatised (Crentsil 2007; Kwansa 2013; Mill 2003; Radstake 2003; Riley & Baah-Odoom 2012; Tenkorang, Gyimah, Maticka-Tyndale & Adjei 2011). In Ghana, reasons for stigmatising HIV/AIDS infection are similar to those in other parts of sub-Saharan Africa (cf. Mbonu *et al*. 2009). HIV is a highly stigmatised condition compared to many other chronic conditions because it is considered to be contagious and severe; and it is assumed to be the result of volitional behaviour, such as commercial sex work or infidelity, which is considered by many to be norm-violating. Neema, Atuyambe, Otolok-Tanga, Twijukye, Kambugu, Thayer, *et al*. (2012) show how moral judgements about sexual behaviour, as well as the language used about HIV, compound the problem of stigma and discrimination.

The fact that HIV prevalence in Ghana is very low compared to, for example, Uganda, where no stratum of society is untouched and every family is affected by the disease (as described by Neema, Atuyambe, Otolok-Tanga, Twijukye, Kambugu, Thayer, *et al*. 2012), means that the disease is less familiar and the stigma associated with it is worse. The way Ghanaians view people with HIV could be encapsulated in the phrase: unknown makes unloved. In this study, PLWHA often experienced shame (*animguaseè*), and loss of respect (*onni buo*) and honour (*ònni animuonyam*) in the eyes of relatives and members of society. These manifestations of stigma might also lead to negative repercussions such as divorce, rejection, ostracisation, discrimination and loss of employment.

Studies across the African continent point out that stigma remains the most prominent factor inhibiting people with HIV or AIDS from seeking health care (Campbell, Skondal & Gibbs 2011; Sengupta, Banks, Jones, Miles & Smith 2011; Stangl *et al*. 2013). Goffman, whose 1963 work on stigma is considered seminal to this topic, defines stigma as an undesirable or discrediting attribute that an individual possesses, thus reducing that individual’s status in the eyes of the community. Stigmatisation is a dynamic process that arises from the perception that there has been a violation of a set of shared attitudes, beliefs and values. HIV/AIDS stigmatisation leads to prejudicial thoughts, behaviours and/or actions on the part of governments, communities, employers, healthcare providers, co-workers, friends and relatives. Stigma can be divided into felt or perceived and enacted stigma (Brown *et al*. 2003). Felt stigma refers to real or imagined fear of societal retribution, and enacted stigma refers to the experience of discrimination. To understand why the clinic has become a safe haven for HIV/AIDS clients in Ghana, it is important to understand the co-producing processes of the two: the experience of and the fear for the destabilising effects of discrimination make people to seek ways to limit the occurrence of enacted stigma. In other words, stigma enhances secrecy and denial.

The invisibility and stigmatisation of HIV/AIDS in Ghana is not conducive to the uptake of voluntary counselling and testing and ART. According to the GHS/NACP (2013), other limiting factors are the out-of-pocket payment required for some services and the generally poor health-seeking behaviour of many Ghanaians. Stigma and lack of finances isolate people from their families and/or communities and affects the overall quality of the life of HIV patients even more. As French *et al*. (2014) argue, it is important to find ways to empower those living with HIV and AIDS to face stigma. In this article, we will argue that the persistent stigma associated with HIV infection makes it imperative, from the point of view of those living with the condition, to secretly use counselling, testing and treatment services. This enables them to conceal their status from relatives and friends and, ultimately, prevent stigmatisation. By choosing the clinic as a suitable place outside their real homes for certain activities, such as praying, they attempted to take control over the consequences of stigmatisation. This article explores how clients made the clinic their home, and it analyses their ability to exercise agency in relation to their own assessment of what the effects might be of either disclosing or not disclosing their HIV status to a wider group of people.

## Actors and activities in the clinic

Health workers in the voluntary counselling and testing centre provided pre- and post-test counselling; and HIV testing is also done in these centres. Clients who tested positive were referred to the ART clinic for counselling in treatment adherence, treatment and supply of antiretroviral medicines. The work of care providers (especially adherence counselling) is complemented by peer educators who are volunteers and also people with HIV. Clients visit the clinics at various periods for a review of their health and to replenish their drugs, depending on what is left of their last supply and their current health status. In this regard, the main actors in the clinic are health workers, peer educators and clients. According to McColgan (2005) one can call a place ‘home’ if one finds intimacy; a home is a place where one is known and feels confident. In this study, some clients had patronised the clinic for more than three years, making monthly visits or at most every six months for the review of their health and the re-supply of medicines. The clinic had thus become a familiar place and the relationships with the health workers had become personal. Since the treatment they receive is lifelong, this relationship between the clients and the clinic is likely to continue for the rest of their lives.

Home is the place where one lives and loves. It can be a location for people needing (professional) care, like a nursing home for the aged. In Ghana, the most basic definition of a ‘home’ means the place where a family lives and carries out most of its daily activities; the physical and providing home is also a social space where family members are nurtured. In a typical Ghanaian home, the family members sharing the house often comprise the parent(s), children and, in many cases, other relatives such as grandparents, uncles, aunts, nieces and nephews. In every home, various groups of people with different roles and responsibilities constitute the inhabitants. Parents take care of children and provide for their needs, while children obey their parents and carry out household chores. Crentsil (2007) notes that among the Akan groups in Ghana, a (family) home is a cherished place where various people live; are always welcome and spend as much time as possible with each other. The adoption and use of the clinic as a ‘home’ for various activities is described below.

### Fictive kin relations

The most noticeable manifestation of the formation of fictive kin relations among the clinic clients was in the manner in which they addressed each other whenever they met. They regularly called each other ‘brother/sister’ (*me nua*) or ‘relative’ (*abusua*). To call someone brother or sister who is not from the same extended family is a powerful way to show affection and concern. Naa, a 26-year-old clinic client, explained that in view of their HIV-positive status and the fact that they have been meeting in the clinic every month, the clients saw themselves as people belonging to ‘one family’, albeit a specific one: a family with a common problem. She said calling each other ‘brother’ and ‘sister’ came naturally as they felt a strong mutual attachment. According to another client, Misaa, they always addressed each other as ‘church member’ (*asòreba*) outside the clinic. Misaa, who was about 33 years old and was also a peer educator, explained that whenever they call each other *asòreba* in public, other people do not understand that they mean by the term fellow-HIV/AIDS clients. Calling each other ‘church member’ similarly enabled them to address each other without exposing their HIV status. It is also a powerful term by which to show solidarity, and it is common in Ghana that members of the same church show solidarity by relating to each other as brothers and sisters.

A similar process happened in a clinic in Uganda. The Creativity Initiative was instituted in a clinic to help reduce HIV-related stigma (Neema, Atuyambe, Otolok-Tanga, Twijukye, Kambugu, Thayer, *et al*. 2012). The initiative was originally intended to provide activities in the clinic while patients waited to be seen by the healthcare professionals; however, it eventually expanded to the learning of art and crafts and training in how to develop entrepreneurial skills. Under the Initiative, patients benefited from social and spiritual support in the clinic. The similarity between the Creativity Initiative and the clinic as a home in the present study is that both are aimed at helping HIV patients cope with stigma through the clinic where they access services. Whereas the Creativity Initiative helped to put patients and staff on more trusting terms and to build the self-esteem of both by establishing a supportive ‘friend’, in this Ghanaian study, the strong bonds of friendship among clients evolved in a more spontaneous way. Although there is a difference between the two processes, with the Ugandan Initiative being more consciously planned in terms of its social objectives, both processes empowered clients to manage their HIV-positive status. The metaphorical usage of family terms empowers PLWHA in Ghana even more, because of the strong kinship ideology (Nukunya 2003).

### Association meeting and counsel

Peer educators were also concerned about the possible exposure of the status of clients when they held meetings at places outside of the clinic or their households, typically public places such as open-air restaurants. Therefore, they performed their role as vigilant ‘uncles’ and ‘aunts’. Kwaa, a peer educator who is about 35 years of age, explained how the clinic had become central to the activities of the various PLWHA associations; it was first of all a safe haven and hence had become the place to find solace and inspiration for many clients. ‘The truth is that the clinic has simply become the home for all of us clients in many respects, except that we do not sleep here’, he stated. In other words, the clinic had become an indispensable part of clients’ lives. Even association members who had stopped attending meetings could not stay away from the clinic forever; sooner or later they would return for a re-supply of drugs. According to Kwaa, peer educators always used the clinic to see clients who had stopped attending association meetings in order to reconnect with them. They also often met association members in the clinic to book appointments for home visits.

For instance, one association moved its meetings from an open-air restaurant to a clinic because it was considered to be a safe venue for clients to freely deliberate on issues related to their HIV-positive status. At one such meeting three people – a peer educator, another client and a nurse – were advising Adobea, aged about 34, not to confront her church authorities about the unfair treatment meted out to her and her husband. They warned her that such an action could let many people know of their positive status with even wider negative implications. They suggested that the couple should leave the church as the only way to avoid further stigmatisation and discrimination. Later when the researcher talked to Adobea, she confirmed that she and her husband had followed the advice and had left the church. Adobea added that they met at the clinic to discuss the matter, rather than at her house, because of the possibility of her neighbours eavesdropping.

Many clients came to the clinic to seek advice about the emotional, social and financial challenges they were facing. They would, for example, ask a nurse for a minute after work, or arrange to see the nurse on a non-clinic day. One client, who believed that engaging the help of a health worker to disclose her positive status prevented the break-up of her marriage, commented as follows: ‘It was this nurse (she mentions the name) who saved me by using an arranged meeting between me, my husband and her and told him that I have this disease (HIV). Else, I would have been divorced by now … ’ (Fauzia, about 28 years of age). Peer educators pointed out that most of the clients had not been able to disclose their status to their spouses and relatives for fear of being divorced, losing respect or being ostracised. Some of these clients sought help from health workers with disclosing their status to their partner. This way they hoped to prevent the possible negative repercussions that could follow if they did the disclosure by themselves. The authority of health workers, especially nurses, enabled them to disclose in a securer way.

### Fraternising

The clinic thus became the place to meet fellow patients to fraternise and share common problems. According to most of the clients, they were able to converse quite openly with peers on a wide range of issues such as treatment outcomes, physical challenges, emotional pain and family problems. Baah, a client of about 40 years old, described his fellow clinic users as ‘sickness family members’ (*yareè abusuafoò*). He said at the clinic he freely helped, interacted and conversed with *yareè abusuafoò* which helps to release some of the stress experienced at home. Baah had many HIV-positive friends with similar problems as his own and they normally met at the clinic to discuss and advise each other on managing their conflicts or difficulties, such as taking their medicine secretly in their family homes. ‘For some of us’, he said, ‘the clinic has become another home besides our original homes where we can find some happiness by laughing over our problems.’

Aba, aged 25 years, said sometimes she came to the clinic without having an official appointment just to hang around and chat with her fellow patients. Before she got infected Aba was a trader travelling with her wares from one town to the other on market days. Before being diagnosed as HIV positive, Aba had been sick for a long time and had spent all her money on treatments outside the hospital. She said she had become fed up with being unemployed and spending most of the time doing virtually nothing. To alleviate the boredom, on non-clinic days she visited some of the nurses who she has taken as her ‘parents’ and friends. She also came to the clinic to speak with her peers about the challenges they are going through, and to share her own experiences. Aba explained that while she did not always take pleasure in listening to the stories of fellow clients, hearing about some of their extreme difficulties made her realise that her own position was not that bad:

Some of the cases they tell me are worse than what I went through before I started treatment here in the clinic. These sometimes serve as encouragement for me to feel that in my case things were a little better and there is still hope for me in life (anidasoò wò hò ma me).

### Settling disputes

According to clients, the way some of the health workers related to them also made the place a ‘home away from home’.^2^ This helped them to forget for a moment the difficulties they were going through. Unlike some of their relatives, most of the health workers showed compassion towards them whenever they visited the clinic. Serwaa, who was about 30 years of age and a trader, explained how some clients have adopted particular nurses as their ‘mothers’ because of their caring behaviour. She always solicited the views of one particular nurse on her private problems besides ARV treatment.

The researcher observed repeatedly how a client would take a nurse as a mother after being treated well. Once a client felt comfortable and safe with a particular nurse, s/he would involve the nurse in personal problems or a dispute s/he may be having with the spouse or a relative. The client would subsequently ask the health worker to advise him or her on how to solve the problem. Health workers usually responded positively and took care to provide sensible advice. The researcher regularly observed cases where the health workers asked the parties involved in the conflict to come to the clinic for a discussion to find a solution. On such occasions, the health workers and the clients with their relatives met at the clinic premises or in the offices of the hospital.

In one instance, nurse Suzane, who had been adopted as a ‘mother’ by Asempa, helped to solve a problem that Asempa had with her husband (who is also HIV positive) and her mother-in-law. Asempa, aged 30, had been harassed and accused by her mother-in-law for being infertile. The mother-in-law threatened that she would get her son to divorce Asempa for failing to give birth to a child. Asempa explained that she had asked her husband to talk to his mother to stop the harassment but that he had failed to do so. Nurse Suzane then invited Asempa and her husband to the clinic for a discussion. She advised the couple to move out of the family house where they lived with the mother-in-law, and she suggested that the husband should explain to his mother that the doctor treating Asempa had advised her not to give birth within the next two years. The nurse advised Asempa’s husband to tell his mother that they were simply following the doctor’s advice to ensure that his wife fully recovers from her health problem. The next time the researcher met Asempa in the clinic, she said that nurse Suzane’s advice had helped to resolve the dispute with her mother-in-law. Asempa explained that they were now living far away from the mother-in-law. She added, ‘Now, I am free from all the insinuations and threats from that old woman.’

It is worth noting that health workers played a very important role in settling disputes between clients in the clinic. Commenting on both the role of health workers in settling disputes and that of the clinic as a convenient venue for this purpose, nurse Benedicta said: ‘Apart from our work as care providers, we also have the additional responsibility of settling disputes in the clinic here because most of the clients are not sincere in disclosing their status to their spouses.’

### Buying and selling

In Ghana, many people have shops or stalls, both called kiosks, in front of their houses, as a way to make an extra income. Petty traders often carry their wares round the community and enter houses to advertise their items. It is also common to see food vendors going round the hospital grounds and entering the ART clinics while verbally advertising the items they are selling, such as pastries and fruits – apples, bananas and oranges. Other items that are sold include clothes, household items, drugs and vegetables. Health workers, peer educators and clients buy and sell in the clinic as they do around their family homes. Some of the clients, who were traders, brought some of their wares to the clinic to sell on both clinic and non-clinic days. Abrefi, about 33 years of age, who sold second-hand clothes, said the health workers in the clinic were some of her most reliable customers. She would select the best of the wares and bring them to the health workers and clinic clients to buy. Virginia, a peer educator who was selling pastries in the clinic, explained how her regular visits to the clinic provided her with a regular group of customers. According to Virginia, aged 32 and mother of 2 children, being a peer educator was her work as she took pride in being part of a larger organisation, and selling pastries was additional work. Moreover, the small profit she got helped her to take care of herself and the children. She remarked: ‘Without this small business I am doing in the clinic, my children and I would not eat. So far as we are all working in the clinic, we have to make a living from here … ’.

Health workers were also selling medicines to clients outside the clinic’s pharmacy, just like drug peddlers do in the community. From our inquiries as to why health workers were not selling those medicines through the clinic’s pharmacy, we found that some health workers were in alliance with pharmacists in town who supplied them with the medicines to be sold to clients directly. These health workers were often given a percentage of the sales as commission. Most of the medicines sold to clients outside the pharmacy were multivitamins, which, according to the health workers, boost the appetite of clients when they take them. One health worker was selling soft drinks such as Coca Cola, Fanta, Sprite and malt to her colleague health workers, clients and their relatives and visitors in the clinic.

The informal trade going on in the ART clinics shows how these places have become integrated into the larger social environment of the hospital. Moreover, it shows the normalisation of the status of HIV and AIDS clients; besides being clients and/or peer educators they had become trustworthy vendors.

### Praise and worship

In many Ghanaian households the day is started with a common prayer to ask for God’s guidance and protection for family members. Similarly, health workers often prayed with clients in the clinic before starting the day’s work. They led clients to sing songs and thank the Lord for taking good care of them. Both health workers and clients prayed that God should protect positive persons from opportunistic infections and provide for their needs.

Interestingly, people from different religious backgrounds, Christians, Muslims and other believers, were all actively involved in these prayers, a level of integration that rarely happens in society at large. During prayers the atmosphere in the clinic became charged and lively, with clients and their accompanying relatives and health workers singing songs of praise to God and clapping their hands in happiness. One particular gospel song that nurse Benedicta used to start the morning devotion with was especially popular. The song was sung in Twi, the lingua franca in most parts of Ghana: *Enti me paa ni na woayè me sè yi, Awurade wo ho yè hu … Woasesa me hyèbrè*,^3^ which literally means, ‘Am I the one you have made like this? God you are wonderful. You have changed my destiny.’ According to nurse Benedicta, for the clients the song meant that

they cannot believe that they are still alive because as positive persons, they would have been dead by now [if not for receiving ARVs]. Since they are still alive, it is by the grace of God who has made this possible through ART treatment.

She explained that with this gospel song, she was encouraging the clients that in the name of God there was hope for them to live longer on treatment. Singing together was an experience that the clients appreciated as an intimate moment of togetherness which gave them solace and Efua, a client aged 26, commented as follows:

The prayer and worship part of care and treatment in this clinic often gives me the hope that I will recover from this disease one day … This is the more reason why I always come to the clinic early so I do not miss this part … it encourages me …

### Choosing a partner

For some people, the clinic was an avenue to find a partner and get married. Many HIV-positive persons were reluctant to disclose their status to their spouses, let alone to potential (marriage) partners. Asiedua, a client aged 24, told of how she met her partner in the clinic and later got married to him. At the time when she tested HIV positive she had told her boyfriend and to her distress he abruptly ended the relationship. She then decided not to enter into any love relationship for fear that she may infect an innocent person. But when Asiedua met Ansah, a male client aged 27, she changed her mind because she felt that she was too young to stay alone. She thought that it would be better to look for a partner who is also positive so she would not infect a lover who is HIV negative.

Asiedua explained how the fact that they were both clients at the same clinic created a strong bond between them and they understood each other’s situation without having to explain it all. Sharing the same predicament, they trusted each other and supported each other in the treatment of the disease. Asiedua said she was grateful to God for making it possible for her to have found a husband in the clinic. Another client, Fremaa, aged 24, also narrated how she met a man in the clinic and eventually got married to him. She was keen to tell her story: a year after having started her treatment she noticed that a male client boarded the same bus with her a couple of times to the clinic. On all those occasions, the two of them got out of the bus at same place and took different routes to the clinic while meeting again in the queue. Without speaking to each other, but having recognised each other, the following visits they alighted from the bus together and walked together to the clinic. After a few times they started talking together and as Fremaa said with a smile, ‘the rest is history’:

So after a while, we conversed briefly in the clinic and got to know each other’s name … On another occasion, when the two of us finished treatment and we were walking to the lorry park, he spoke to me and then fell in love with me. Now, we are happily married as husband and wife. This is my love story in the clinic …

## Discussion

The discussion looks at three issues. It starts with an analysis of why the family homes of clients were no longer experienced as homes, or have lost their character as homes, then it analyses what made the clinic a home for clients, and finally, it examines how the fictive kin relations established in the clinic can be seen as the therapy management group.

The way that clients used the clinic as a home suggests that anywhere can be made into a home provided that it is comfortable to identify with the particular place. However, as McColgan (2005) says, for a place to be felt to be a home, it must have certain basic characteristics. Most important, a home must guarantee the privacy and safety, in short trust, of the individual so they can go about certain aspects of their life unnoticed by all except those whom they consider to be insiders. A home should also be a place where one can experience love and affection from the other members and one must feel comfortable and secure. Home is the place where one interacts intimately with familiar ones – sharing food, watching TV, finding a listening ear, doing the laundry or exchanging views. It is a place of refuge where one can find solace and peace in difficult times. However, this is an idealised view; in reality there are degrees of being ‘at home’ in one’s home, and homes can sometimes be hostile places where one does not feel safe, or is neglected or abused. Nonetheless, the basic concept of a home is that of a place of comfort, care, privacy and safety and it is this sense of home that the clients in this study have transferred to the clinic environment.

The need for the clinic as a home from home has much to do with the persistent stigma associated with HIV infection. Most of the clients in this study were unable to engage in certain activities within their own home communities (such as using close relatives as their therapy managers) for fear of being exposed as an HIV-positive person. Since the majority of the clients had not disclosed their HIV status even to their immediate family, they did not feel safe to discuss any issues associated with the disease, whether it was their daily medication or inexplicable diseases that were in fact related to their HIV infection. For this reason their homes no longer guaranteed them the privacy and safety that they needed to talk about their health. Some of the clients in the study who did disclose their HIV status to their family lost the love and affection of their family members and were accused of suffering from an ‘immoral’ disease, while others were divorced or rejected by their spouses.

Since many clients lived in fear of inadvertently revealing their HIV status they no longer felt at liberty to converse freely, or do whatever they wanted. They therefore went about their daily chores in an inhibited manner, watchful and tense. This meant that their family homes were no longer places of refuge where they could feel supported to face the difficulties and challenges associated with HIV infection. In essence, they did not feel at home in their family homes and, as a result, they (unconsciously) looked for another place where they would feel safe. According to the clients, the clinic was the place par excellence where they felt at home, rather than, for example, the church, which is for most Ghanaians a ‘second home’. The compassionate attitude and professional practices of health workers made the clinic patient-friendly.

In their work on stigmatised cancer patients in a United Kingdom hospital, Wilson and Luker (2006) also argue that health workers can make a crucial difference for patients. They are inspired by Goffman’s (1963) work on stigma, which he defines as an attribute, behaviour or reputation which is socially discrediting in a particular way; stigma causes an individual to be mentally classified by others in an undesirable, rejected stereotype rather than in an accepted, normal one. Wilson and Luker show that people affected by cancer found a support group which consisted of persons who shared their stigma, and who gave them ‘moral support’ and made them feel ‘at home’. Whereas the outside world was ‘a civil place’ (in Goffman’s terms ‘front stage’) where people affected by cancer were sometimes treated as if they did not qualify for automatic acceptance as people, the hospital turned out to be a ‘back place’ (in Goffman’s terms ‘back stage’), by which the authors meant a place outside of the public eye. Similarly, in a study in the United Kingdom, Hodgson (2006) observed that a unit providing HIV care and treatment was perceived by respondents as especially adept at protecting PLWHA from stigma, and was seen as a place where carers were open – ‘unfazed by unusual or novel situations’ (2006:267). In Goffman’s terms clinic health workers of this kind can be considered ‘the wise’ (1963). Those labelled as ‘sensible’ or ‘wise’ are usually close family members and other supportive associates who are knowledgeable and sympathetic towards people who bear a ‘courtesy stigma’; a stigma acquired as a result of being related to a person with a stigma. Likewise, the supportive attitudes of health workers towards clients in our study made them the ‘wise’. Wise people are generally well respected in Ghana and from the clients’ perspective the health workers behaved as wise people: they act in a confident and comfortable manner during interactions in the clinic.

Another factor contributing positively to the clinic becoming a home was the fact that it is a public space. For the clients, accessing the clinic was not problematic because they could simply be seen as one of the numerous general hospital patients, or even as a visitor to the hospital. They could enter the hospital grounds and for that matter the clinic relatively free of the fear of being recognised specifically as an HIV/AIDS patient. Moreover, as frequent visitors, or lifelong clients, the clinics became familiar ground, and they became familiar faces to the health worker community. This sense of being free to walk in and out made them feel at ease: even though it was a public space it had the attributes of privacy and familiarity that they would find in a private home.

In the clinic, clients found health workers, peer educators and fellow clients to whom they could relate as (fictive) family members. In Goffman’s terms, the fellow clients (hence also the peer educators) can be referred to as the patients’ ‘own’ (1963). Stigmatised individuals view those who share their particular stigma as their ‘own’; they belong to the same in-group, in contrast to those who are ignorant of, if not hostile towards, HIV/AIDS. Experiencing severe stigma created a strong sense of solidarity among the clients, and health workers sympathised with that. Therefore, with the help of ‘the wise’, and their ‘own’, clients created a place and the space to live a life; to fraternise, share worries, seek advice and settle disputes. These positive persons also experienced love and affection from health workers and their peers, something which they had not in some instances experienced for a long time. In fact, the clinic served as a temporary refuge from the problems they went through in their family homes.

Ultimately, use of the clinic as a home has made it possible for clients to form their ‘therapy management group’ (Janzen 1987) within the facility, as mentioned in the introduction to this article. The concept of a therapy management group was coined by Janzen and inspired by his study of health-seeking behaviour among the BaKongo of Lower Zaire.^4^ He characterises the therapy management group as a set of close kin group members who care for the sick person. A group of this nature works on the principle that whenever an individual or a set of individuals becomes ill or is confronted with overwhelming problems, various maternal and/or paternal kinsmen, and occasionally their friends and associates, rally for the purpose of sifting information, lending moral support, making decisions and arranging details of therapeutic consultation (1987:68). Similarly, Bossart (2003), who studied the role of social networks in times of illness in Cote d’Ivoire, observed that the main source of assistance in response to affliction was the household members. Apart from emotional and moral support, relatives outside the household and non-kin, such as friends, also played a role in the care and treatment of the sick person. They often provided financial support to the patient.

The notion of a therapy management group can be extended to many African societies. It is not uncommon in sub-Saharan African countries that family members accompany patients to healthcare settings to care for, cook and wash the patients (cf. Gruskin, Ahmed & Ferguson 2008). Thus, the group’s responsibility goes beyond popular and folk medicine and extends to the hospital setting. Ward environments and clinics tend to be characterised by over-crowding and lack of space for private discussions (see also Mulemi 2010). For most conditions, members of the group will know what type of sickness or disease the patient is suffering from and will thus be in a position to make appropriate decisions about treatment, either in the hospital or at home.

The peculiar nature of HIV/AIDS brings a new dimension to the notion of a therapy management group. In Ghana, normally the therapy management group consists of relatives. However, in the case of HIV/AIDS, where a positive status carries grave social risks (divorce, rejection, ostracism and discrimination) and where hiding one’s status from relatives, friends and neighbours has become a way of life, it is difficult and sometimes impossible for clients to form a therapy management group. In the absence of conventional support these HIV-positive people have found an alternative group in which to confide and which can help them take decisions regarding their lifelong infection and its treatment as well as on personal, emotional and financial matters. As a result of feeling at home in the clinic, clients started using health workers, peer educators and some of their colleagues as members of their therapy management group. They would explain their particular problem, ask for other clients’ experiences and consult those who had been taking ARVs for a longer period. In this new-found home, health workers were adopted as ‘parents’, mostly nurses became ‘mothers’, who helped them to take decisions on treatment and marital problems, while peer educators became the ‘uncles’ and ‘aunts’ who advised and assisted where needed. Clients shared their feelings and concerns on a range of issues with their fellow patients as ‘siblings’. Health workers agreed to be members of clients’ therapy management group because they were aware that the fear of stigma made it difficult for most clients to discuss problems related to their status with relatives. In short, a new therapy management group had been formed.

The decision of clients to form their therapy management with actors in the clinic can be explained as the failure of the concept of ‘indigenous cultural and family insurance’ (Agyemang 2013). As he explains, in most collectivist cultures, an indigenous form of insurance has existed long before the introduction of the biomedical system. The family functioned as a kind of insurance in the form of social support in times of need, such as illness. The concept of insurance must not only be seen as a family responsibility but as part of cultural heritage; it has become a way of life. In Ghana, it is common that family members, neighbours and friends provide social support to people in the event of illness, unemployment, old age or death (Agyemang 2013). However in the case of HIV/AIDS, its persistent stigma has made it difficult for HIV/AIDS patients to inform relatives and even spouses about their status, thus preventing them from providing social support.

In effect, clients’ initiative and decision to make health workers and others in the clinic members of their therapy management group precluded their relatives from being informed about their condition, and excluded them from making decisions and sharing responsibilities related to their condition. As a result of the clients’ non-disclosure of their HIV status to their family and community members, and their adoption of an alternative community where they can receive care behind the scenes, it remained possible for the relatives of the infected person to escape the stigma of being associated with an HIV-positive family member. In Goffman’s (1963) terminology, the family can thus remain the ‘normals’ and be free from the stigma of being associated with an infected family member, the ‘courtesy stigma’, and its negative consequences. This reality was borne out over and over again in the experience of the clients in this study: fearing the effects of stigma, they shunned those whom they would normally turn to for help. The non-disclosure of people’s HIV status meant that relatives and household members remained ignorant about the disease and its treatment, thus preventing them from ever becoming suitable as therapy group members. Fear of stigma thus prevented clients from adopting relatives as members of their therapy management group. This role was restored in the clinic environment, demonstrating how health workers and clients mutually constitute an alternative to common health-seeking behaviour in cases where the family is not considered to be supportive.

## Conclusion

HIV/AIDS clients maintain a lifelong relationship with, mostly, the same clinic, its health workers and their fellow clients. This study has shown how clients used the clinic as their second home. The small community that the clinic provided became a familiar place and a safe haven, where clients and other agents created enduring relationships, as if they were a family. These relationships can be seen as fictive kin, who in this case assume part of the role that kin relations provide, forming the therapy management group. The paralysing fear of stigma made clients turn for support to those with whom they felt safe and whom they trusted. They felt that the clinic, unlike their personal homes, guaranteed them the privacy they needed to live life as an HIV-positive person. In effect, they were able to prevent people in their family homes from knowing their status. This was a logical desire, as it helped them to maintain their position, respect and reputation within their families and community. The most dramatic outcome of this understandable desire was that, contrary to Ghanaian norms and values, people turned to non-kin for assistance. We have described how they formed alternative therapy management groups, which, in a dire situation, can be seen as an encouraging outcome despite the fact that it also underscores, once more, the predicament in which many PLWHA find themselves.

In short, the conclusion of this study is that the ART and VCT services studied in this research are successful in terms of providing health care and hence enhancing adherence. The drawback is that this success hinges upon another painful conclusion that PLWA do not disclose their status to close relatives. Yet, we believe that the successful interventions of the ART and VCT centres are an important starting point to think further about creating space for PLWA to enable them to disclose to their family and friends. Creating safety is crucial and this study shows how two clinics in a highly stigmatising context managed to do so. Compassion is a crucial element of the professionalisation of healthcare workers and in the up-scaling of HIV/AIDS programmes in low-prevalence countries, it is important to invest in this aspect of healthcare provision.

## Notes

1. HIV-positive patients who access treatment in the clinics are referred to as ‘clients’ in this study, or sometimes as ‘positive persons’.

2. The study revealed that with a few exceptions health workers providing VCT and ART services related to and treated clients well. They mostly displayed good practices towards clients during clinical encounters. For instance, they warmly received clients, they advised clients on a wide range of issues including marital and family problems, and comfortably interacted with clients. This led to lasting relationships between the two groups, particularly nurses and clients.

3. The song paraphrased in Twi language is by Pastor Joe Becham, a Ghanaian gospel musician.

4. Zaire is now called Democratic Republic of Congo.

## References

[CIT0001] Agyemang B. C. (2013). HIV/AIDS Stigmatization on Relations and Associates of People Living with HIV/AIDS: A Psychological Study. International Journal of Health and Psychology Research, 1(2), 1–13.

[CIT0002] Andersen H. M. (2004). ‘Villagers’: Differential Treatment in a Ghanaian Hospital. Social Science and Medicine, 59, 2003–2012. doi: 10.1016/j.socscimed.2004.03.005 15351468

[CIT0003] Bossart R. (2003). ‘In the City, Everybody Only Cares for Himself.’ Social Relations and Illness in Abidjan, Cote d’Ivoire. Anthropology and Medicine, 10(3), 343–359. doi: 10.1080/1364847032000133852 26954987

[CIT0004] Brown L., Macintyre K. & Trujillo L. (2003). Interventions to Reduce HIV/AIDS Stigma: What Have We Learned? AIDS Education and Prevention, 15(1), 49–69. doi: 10.1521/aeap.15.1.49.23844 12627743

[CIT0005] Campbell C., Skondal M. & Gibbs A. (2011). Creating Social Spaces to Tackle AIDS-Related Stigma: Reviewing the Role of Church Groups in Sub-Saharan Africa. AIDS and Behaviour, 15(6), 1204–1219. doi: 10.1007/s10461-010-9766-0 PMC3514979 20668927

[CIT0006] Crentsil P. (2007). Death, Ancestors, and HIV/AIDS among the Akan of Ghana. PhD dissertation, University of Helsinki.

[CIT0007] Dapaah J. (2012). HIV/AIDS Treatment in Two Ghanaian Hospitals. Experiences of Patients, Nurses and Doctors, Leiden, African Studies Centre.

[CIT0008] French H., Greeff M. & Watson M. (2014). Experiences of People Living with HIV and People Living Close to Them of a Comprehensive HIV Stigma Reduction Community Intervention in an Urban and a Rural Setting. SAHARA-J: Journal of Social Aspects of HIV/AIDS, 11(1), 105–115. doi: 10.1080/17290376.2014.938104 25019454 PMC4272103

[CIT0009] Ghana Health Service (GHS)/National AIDS Control Programme (NACP) (2013). 2013 HIV Sentinel Survey Report, Accra, Ghana.

[CIT0011] Goffman E. (1963). Stigma: Notes on the Management of Spoiled Identity, Englewood Cliffs, NJ, Prentice Hall.

[CIT0012] Gruskin S., Ahmed S. & Ferguson L. (2008). Provider-Initiated HIV Testing and Counselling in Health Facilities: What Does This Mean for the Health and Human Rights of Pregnant Women? Developing World Bioethics, 8(1), 23–32. doi: 10.1111/j.1471-8847.2007.00222.x 18315722

[CIT0013] Hodgson I. (2006). Empathy, Inclusion and Enclaves: The Culture of Care of People with HIV/AIDS and Nursing Implications. Journal of Advanced Nursing, 55(3), 283–290. doi: 10.1111/j.1365-2648.2006.03913.x 16866822

[CIT0014] Janzen J. M. (1987). Therapy Management: Concept, Reality, Process. Medical Anthropology Quarterly, 1(1), 68–84. doi: 10.1525/maq.1987.1.1.02a00040

[CIT0015] Jewkes R., Abrahams N. & Mvo Z. C. (1998). Why Do Nurses Abuse Patients? Reflections from South African Obstetric Services. Social Science and Medicine, 47(11), 1781–1795. doi: 10.1016/S0277-9536(98)00240-8 9877348

[CIT0016] Kwansa B. K. (2013). Safety in the Midst of Stigma. Experiencing HIV/AIDS in Two Ghanaian Communities, Leiden, African Studies Centre.

[CIT0017] Mall S., Middelkoop K., Mark D., Wood R. & Bekker L.-G. (2013). Changing Patterns in HIV/AIDS Stigma and Uptake of Voluntary Counselling and Testing Services. AIDS Care, 25(2), 194–201. doi: 10.1080/09540121.2012.689810 22694602

[CIT0018] Mbonu N., Van Den Borne B. & De Vries N. (2009). Stigma of People with HIV and AIDS in Sub-Saharan Africa: A Literature Review. Journal of Tropical Medicine, 2009, 1–14. doi: 10.1155/2009/145891 PMC2836916 20309417

[CIT0019] McColgan G. (2005). A Place to Sit: Resistance Strategies Used to Create Privacy and Home by People with Dementia. Journal of Contemporary Ethnography, 34, 410–433. doi: 10.1177/0891241605275574

[CIT0020] Mill I. (2003). Shrouded in Secrecy: Breaking the News of HIV Infection to Ghanaian Women. Journal of Transcultural Nursing, 14, 6–16. doi: 10.1177/1043659602238345 12593265

[CIT0021] Mulemi A. (2010). Coping with Cancer and Adversity: Hospital Ethnography in Kenya, Leiden, African Studies Centre.

[CIT0022] Neema S., Atuyambe L. M., Otolok-Tanga E., Twijukye C., Kambugu A., Thayer L., et al. (2012). Using a Clinic Based Creativity Initiative to Reduce HIV Related Stigma at the Infectious Diseases Institute, Mulago National Referral Hospital, Uganda. African Health Sciences, 12(2), 231–239. 23056033 10.4314/ahs.v12i2.24 PMC3462528

[CIT0023] Nukunya G. (2003). Tradition and Change in Ghana: An Introduction to Sociology, Accra, Ghana Universities Press.

[CIT0024] Radstake M. (2003). Secrecy and Ambiguity: Home Care for People Living with HIV/AIDS in Ghana, Leiden, African Studies Centre.

[CIT0025] Riley G. A. & Baah-Odoom D. (2012). Belief in a Just World, Generalized Self-efficacy and Stigma May Contribute to Unsafe Sexual Intentions Via a Reduced Perception of Vulnerability to HIV/AIDS Amongst Young People in Ghana. AIDS Care, 24, 642–648. doi: 10.1080/09540121.2011.630348 22087574

[CIT0026] Sengupta S., Banks B., Jones D., Miles M. S. & Smith G. C. (2011). HIV Intervention to Reduce HIV/AIDS Stigma: A Systematic Review. AIDS Behaviour, 15(6), 1075–1087. doi: 10.1007/s10461-010-9847-0 PMC3128169 21088989

[CIT0027] Spronk R. (2011). Promoting Access to and Use of Voluntary Counselling and Testing and Antiretroviral Therapy among People Living with AIDS in Ghana. A Multi-level Study, Project report, University of Amsterdam.

[CIT0028] Stangl A., Lloyd J., Brady L., Holland C. & Baral S. (2013). A Systematic Review of Interventions to Reduce HIV-Related Stigma and Discrimination from 2002 to 2013: How Far Have We Come? Journal of the International AIDS Society, 16(3 Suppl 2), 18734. 24242268 10.7448/IAS.16.3.18734 PMC3833106

[CIT0029] Tenkorang E., Gyimah O., Maticka-Tyndale E. & Adjei J. (2011). Superstition, Witchcraft and HIV Prevention in Sub-Saharan Africa: The Case of Ghana. Culture, Health and Sexuality, 13(9), 1001–1014. doi: 10.1080/13691058.2011.592218 21714753

[CIT0030] Wilson K. & Luker A. K. (2006). At Home in Hospital? Interaction and Stigma in People Affected by Cancer. Social Science & Medicine, 62, 1616–1627. doi: 10.1016/j.socscimed.2005.08.053 16198466

[CIT0031] Wind G. (2008). Negotiated Interactive Observation: Doing Fieldwork in Hospital Settings. Anthropology & Medicine, 15(2), 79–89. doi: 10.1080/13648470802127098 27269225

[CIT0032] Van Der Geest S. & Finkler K. (2004). Hospital Ethnography: Introduction. Social Science and Medicine, 59, 1995–2001. doi: 10.1016/j.socscimed.2004.03.004 15351467

[CIT0032a] Van der Geest S. & Sarkodi S. (1998). The Fake Patient: A Research Experiment in a Ghanaian Hospital. Social Science and Medicine, 47, 1373–1381.

